# Molecular epidemiology of Eimeria spp. parasites and the faecal microbiome of Indiana bats (Myotis sodalis): a non-invasive, multiplex metabarcode survey of an endangered species

**DOI:** 10.1099/mgen.0.001358

**Published:** 2025-02-26

**Authors:** Andrew J. Bennett, Cory D. Suski, Joy M. O’Keefe

**Affiliations:** 1 Department of Natural Resources and Environmental Sciences, University of Illinois at Urbana-Champaign, Champaign, IL, USA

**Keywords:** 16S microbiome, 18S parasitome, *Clostridium*, *Eimeria*, molecular epidemiology, multiplex metabarcode sequencing

## Abstract

Assessing individual and population health in endangered wildlife poses unique challenges due to the lack of an adequate baseline and ethical constraints on invasive sampling. For endangered bats, minimally invasive samples like guano can often be the ethical and technical limit for studies of pathogens and the microbiome. In this study, we use multiplex metabarcode sequencing to describe the faecal microbiome and parasites of 56 Indiana bats (*Myotis sodalis*). We show evidence of a high prevalence of *Eimeria* spp. protozoan parasite and characterize associations between infection and changes to the faecal microbiome. We identify a strong and significant enrichment of *Clostridium* species in *Eimeria*-positive bats, including isolates related to *Clostridium perfringens*.

**Impact Statement**

A central challenge in wildlife health is assessing the impact of disease on protected, non-model organisms. For domesticated or unprotected species, health can be assessed via regular invasive sampling of housed individuals, or more frequent lethal sampling of tissues for ‘gold standard’ pathology methods. However, for cryptic and endangered wildlife like the Indiana bat (*Myotis sodalis*), routine monitoring is impossible, and extensive lethal sampling is unethical. In this study, we use multiplex metabarcode sequencing to identify associations between the faecal microbiome and infection with an *Eimeria* species coccidian gut parasite in Indiana bats. We describe the Indiana bat faecal microbiome and identify associations between *Eimeria* and microbiome changes. We characterize the expansion of diverse *Clostridium* spp. bacteria in *Eimeria*-infected bats, representing clades typically associated with necrotizing enteritis in other species. Viewing these connections through a comparative biomedical lens, this study represents a non-invasive approach to evaluating potential threats to the population health of endangered species or hosts for which there is limited baseline surveillance data.

## Data Summary

Reads are available as a BioProject with cross-identified BioSamples for each gene target and sequence read runs (SRR) in a sequence read archive (SRA) at the National Center for Biotechnology Information GenBank (BioProject: PRJNA1092112; SRR/SRA accession numbers in Supplementary Material; ‘Data Summary’). The amplicon sequence variant dataset is also available as DADA2 outputs for phyloseq at the Illinois Data Bank of the University of Illinois Urbana-Champaign (https://doi.org/10.13012/B2IDB-3079533_V1).

Table S1, available in the online version of this article: SRA data summary. Summary of National Center for Biotechnology Information GenBank SRR included in this study (*N*=114). Each SRR represents the 16S microbiome or 18S parasitome metabarcode sequencing results for one Indiana bat faecal BioSample (*N*=57).

## Introduction

Assessing individual and population health in wildlife is challenging, particularly for non-model organisms like bats (order Chiroptera). For protected species, non-lethal, minimally invasive sampling is strongly preferred by management agencies and often the practical limit for ethical infectious disease surveillance of a sufficient sample size [1]. Health is not just the absence of disease, but rather the capacity of populations and individuals to robustly maintain homeostasis in the face of changing environments [2]. Conversely, infectious disease should not be simply defined as the presence of a potential pathogen [3,4]. Molecular surveillance of wildlife can reveal potential pathogens [5], but the detection of a micro-organism may be misinterpreted as an indicator of disease without evidence of pathology [6,7].

Animal microbiomes reflect organismal and ecosystem health and can be an early indicator of disturbances to either [8]. Healthy hosts depend upon their microbiota to maintain homeostasis [2], and because host-associated microbes are active in diverse metabolic pathways for digestion, nutrition and immune function [8], changes to microbial communities can also indicate underlying disease [9]. Finding molecular evidence of pathology – as distinct from merely noting the presence or absence of a pathogen – can be an effective middle ground between sampling tissues for histopathology and drawing epidemiological links between pathogen detection and stress [6,10,12]. Multiplex metabarcode methods can survey population-level variation in the faecal microbiome and eukaryotic parasites in parallel [11] and can identify associations, which are informative of the underlying physiology [12].

In this study, we use a metabarcode sequencing approach to assess Indiana bat (*Myotis sodalis*) health and disease during the fall swarm period. We survey the 16S rRNA faecal microbiome and eukaryotic parasites (18S rRNA) in the guano [11]. This effort represents the first large-scale, next-generation sequence-based molecular survey for micro-organisms ever undertaken for Indiana bats. We describe the faecal microbiome of 56 individuals, which, at a survey level, appears broadly convergent with that of other insectivorous vespertilionid bats [13,14]. We also detected a high prevalence of an *Eimeria* species parasite, correlated with an increase in *Clostridium sensu stricto* 1 bacteria faecal abundance. We characterize the expansion of *Clostridium* in *Eimeria*-infected Indiana bats and a reduction in commensal *Clostridia* [15], which represent a large fraction of *Bacillota* taxa in the species. Viewing these connections through a comparative biomedical lens, we propose this parallel over-expression as a potential molecular biomarker of gut epithelium stress in Indiana bats incorporating evidence from model *Eimeria* research systems. We discuss molecular signatures of microbial interactions and how they can indicate pathology from high-quality, population-level samples collected non-lethally.

## Methods

### Collection of samples

On two nights in Autumn 2022, 57 Indiana bats were collected at the entrance of Missouri hibernacula, using mist nets on 27 September at Lime Kiln Mine (*n*=31; Hannibal, MO, USA) and a harp trap on 6 October at Pilot Knob Mine (*n*=26; Ironton, MO, USA), respectively. Bats gather at these two hibernacula to swarm and mate prior to hibernation. Bats were captured on exit, and samples were collected from bats on a single evening at each site. Nets and traps were continuously monitored, and bats were held individually in single-use mesh drawstring bags and processed within 30 min of capture. Guano was collected from individuals using sterile forceps from holding bags – or directly from bats if excreted during handling – and placed in sterile 2 ml tubes preloaded with analytical grade Supelco® silica gel desiccant (Sigma-Aldrich, Bellefonte, PA, USA). Samples were transported on ice and stored at −20 °C within 6 h of collection. Species-level identification of bats was performed by experienced, permitted team leaders. Prior to release, bat forearms were banded using uniquely numbered, lipped, aluminium bands (2.9 mm; Porzana Ltd., East Sussex, UK), which prevented resampling.

### Faecal metabarcode sequencing for microbiota and parasites

In the laboratory, total DNA was extracted from a single guano pellet (~10 mg) per bat using *Quick*-DNA Fecal/Soil Microorganism Miniprep Kits (Zymo Research Corporation, Irvine, CA, USA). DNA extracts were initially quantified using the NanoDrop system and diluted as necessary to a stock concentration of 10–50 ng ul^−1^. Extracted DNA was submitted to the Roy J. Carver Biotechnology Center at the University of Illinois Urbana-Champaign for multiplex metabarcode library preparation and sequencing. Prior to library preparation, DNA was quantified using the Qubit dsDNA High Sensitivity kit, and samples were normalized to a standard loading concentration for the Fluidigm Access Array (Standard BioTools, San Francisco, CA, USA). On the Access Array, 16S and 18S sequence libraries are generated using microfluidic PCR to parallelize their amplification from a single DNA isolate [16,18]. Parasitome libraries were generated using primers targeting the V3–V4 hyper-variable region of the 18S rRNA of eukaryotic parasites (primers G3F1/G3R1) [11]. For the microbiome library amplification, we used a modified 341F/806R universal prokaryotic 16S rRNA primer set described by Krogsgaard *et al*. [11]. Amplicon libraries were sequenced on the NovaSeq 6000 next-generation sequencing platform (Illumina, San Diego, CA, USA) with 2×250 nt chemistry using standard methods.

### Pre-processing of paired sequence reads

Raw reads were checked for quality using FastQC [19] (version 0.11.5) and trimmed and filtered (>Q30) using DADA2 (version 1.26.0) in R [20]. DADA2 learnErrors method was used to estimate the sequence error rates for the forward and reverse reads. We then pooled, denoised, dereplicated and merged sample reads using DADA2 and removed chimaeras using DADA2’s implementation of removeBimeraDenovo.

### Taxonomic assignment of amplicon sequence variants

Unique, dereplicated amplicons were binned as amplicon sequence variants (ASVs) and assigned taxonomy via DADA2’s naïve Bayesian Ribosomal Database Project (RDP) classifier in R (minboot=50) [20,21]. DADA2 trains a machine learning algorithm using a well-resolved reference tree generated from curated 18S or 16S ribosomal databases (silva 18S v132; silva 16S v138) [22] to classify ASVs and test the confidence of the taxonomic assignment [20]. Multiple sequence alignments for the RDP classifier were performed using DECIPHER [23] (version 2.14.0). Midpoint-rooted, maximum-likelihood phylogenies were reconstructed for ASVs of each sequencing primer set using Phangorn [24] (version 2.5.5) and ape [25] (version 5.3) packages in R.

Additional sequence homology searches using blastn [26] (version 2.15.0+) were performed for specific ASVs. These were performed first against the National Center for Biotechnology Information GenBank’s 16S rRNA (Bacteria and Archaea type strains) reference database and then against core_nt (databases updated: 8 February 2024) if no curated type strains shared at least 90% homology with the ASV. The taxonomic classification for ASVs is dependent upon the Bayesian classifier previously described unless otherwise noted. For each primer-sorted set of quality-filtered and binned ASVs, a phyloseq object was generated using the phyloseq package (version 1.38.0) [27] in R [28].

### 16S and 18S metabarcode dataset filtering and normalization

In phyloseq, taxonomic filtering of 16S datasets (*n*=57) removed ASVs not assigned to Bacteria and off-target amplicons of organellar DNA (e.g. mitochondria and chloroplast). By filtering the data, we removed low-prevalence taxa only present in extremely low abundance (≤3 reads in <5% of samples) [29]. Closely related taxa were agglomerated using tip_glom with agglomerative nesting (*H*=0.1) [27]. 16S samples were rarefied to 100 k reads (set.seed=40), which pruned one sample from downstream analyses. Krona plots were generated using phyloseq’s plot_krona function. 18S rRNA datasets (*n*=56) were rarefied to 10 k reads, with taxonomic filtering to include only members of the *Stramenopile*, *Alveolate*, and *Rhizarian* (SAR) supergroup. Taxa were agglomerated using tip_glom as described above. Normalized abundances of taxa, where referenced, represent the proportion of reads assigned to a taxa in the rarefied datasets.

### Determination of *Eimeria* infection status

For our determination of *Eimeria* infection status, we observed the total abundance of *Eimeria* spp. reads in our normalized 18S dataset. Samples were only considered *Eimeria*-positive if *Eimeria* genus reads represented at least 0.1% of rarefied reads (normalized abundance ≥100/100 000) [29]. The mean difference in normalized abundance of *Eimeria* spp. reads between our *Eimeria-*positive (*n*=24) and *Eimeria*-negative (*n*=32) bat groups was 12784.33±6999.65 [95% CI (5784.68, 19783.98)]. In the negative group, the mean abundance of *Eimeria* reads was below the threshold of detection by an order of magnitude [M=6/100 000 reads, 95% CI (−0.82, 12.82)].

For the phylogenetic representation of the complete faecal parasitome of all Indiana bats in our dataset, we agglomerated SAR supergroup eukaryote ASVs by family using tax_glom in phyloseq, and we transformed read counts to relative abundance. We then generated an 18S phylogeny using plot_tree with ggplot2 (version 3.5.0) [30] and phyloseq.

### Differential abundance of microbes in *Eimeria-*positive bats

We used edgeR (version 3.34.1) [31] to assess differential expression in order to identify differentially abundant microbes (DAMs) in the guano of *Eimeria*-positive vs. *Eimeria*-negative bats. For each ASV, we calculated the log c.p.m., log_2_ fold-change and the Benjamini-Hochberg-adjusted *P*-values (*α*=1×10^−6^) to control the false discovery rate.

## Results and discussion

### 16S microbiome composition of Indiana bats, Missouri, Fall 2022

Prior to filtering and normalization, our 16S metabarcode dataset (*n*=57) included 16 349 079 reads representing 33 890 unique ASVs, with a mean of 286 826±76 419 (se) reads and 1159±75 (se) ASVs per sample. We filtered out reads assigned to off-target taxa, including non-bacterial ASVs (1363 reads; 25 ASVs) and sequences derived from chloroplast (226 836 reads; 159 ASVs) and mitochondrial (18 613 reads; 63 ASVs) DNA. Filtering out rare, low-abundance taxa limited the dataset to 2930 ASVs [477±27 (se) ASVs per sample], but per sample, the average number of reads remained high [253 065±75 008 (se)]. Tip agglomeration streamlined the dataset to 672 taxa [mean 155±54 (se) taxa per sample], and no agglomerated taxa were lost to the complete dataset with rarefaction to 100 k reads per sample [mean 141±54 (se) taxa per sample].

**Fig. 1.**
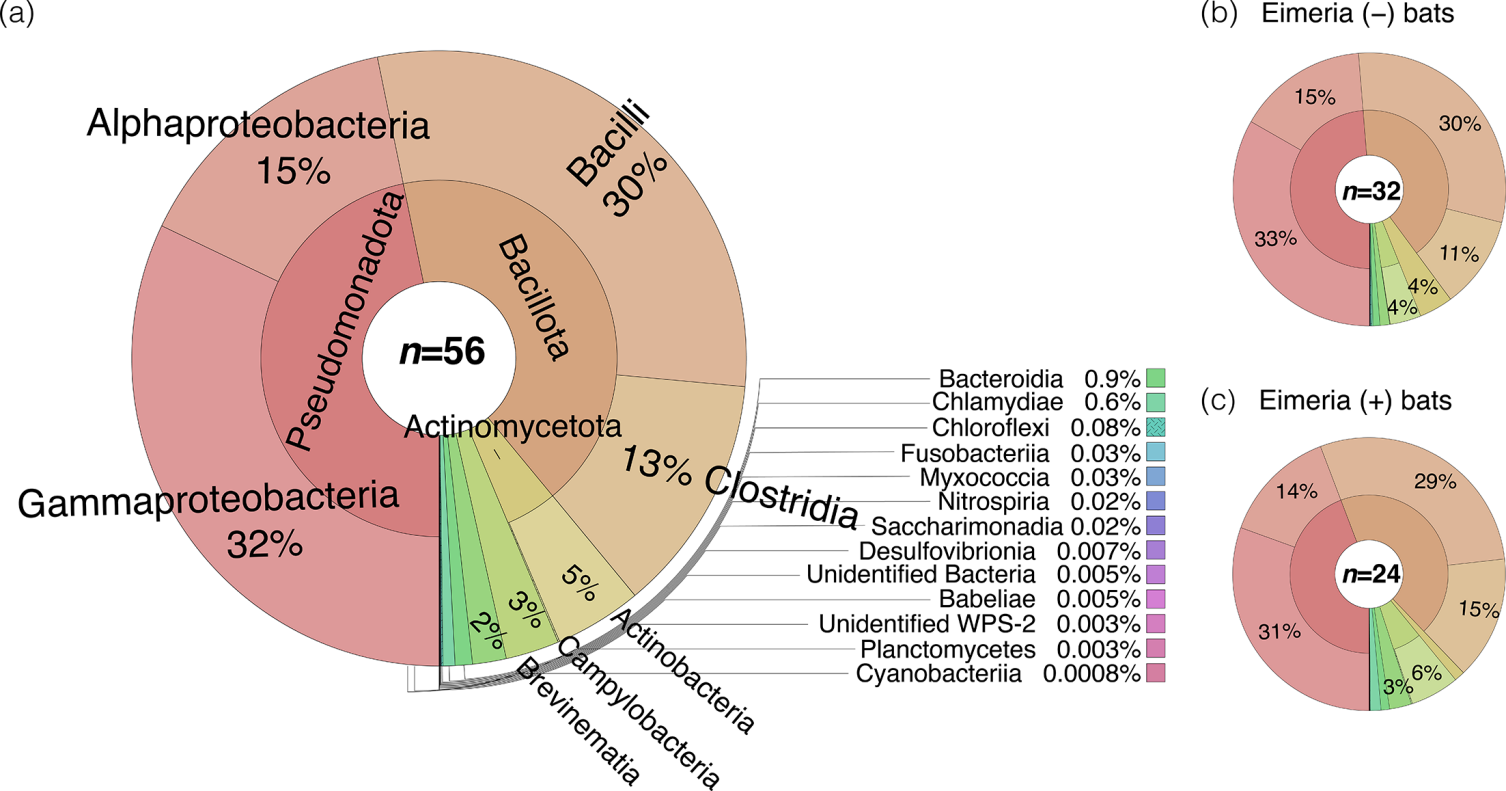
16S faecal microbiome of Indiana bats (*n*=56) aggregating at winter hibernacula in Missouri, Fall 2022. (a) Krona plot of the faecal microbiome composition (to taxonomic class) and relative read abundance of microbes in all Indiana bats in this study. (**b) **Krona plot of the faecal microbiome composition (to taxonomic class) and relative read abundance of microbes in *Eimeria-*negative bats. (**c**) Krona plot of the faecal microbiome composition (to taxonomic class) and relative read abundance of microbes in *Eimeria-*positive bats.

We detected a faecal microbiome dominated by the phyla Pseudomonadota and Bacillota, with Actinomycetota as the distant third-ranked phylum (Fig. 1a). The top 2 phyla match those detected for insectivorous bats in previous studies of the faecal microbiome, with a high proportion of Pseudomonadota (47%; 15% alpha- and 32% gammaproteobacteria) and Bacillota (43%; 30% Bacilli and 13% Clostridia) reads [13,14, 32,34]. As seen previously in bats, there was a low abundance of the *Bacteroides*, which are common to most mammal gastrointestinal tracts, likely due to bats’ short and simple guts with few niches for anaerobes [13,14, 32,34]. As with flying birds, bat intestines are dramatically shorter and simpler than those of their non-volant terrestrial relatives, reflecting the unique constraints of flight [32].

The bat’s large intestine is typically limited to a descending colon, and the transit time of gut contents is short, resulting in a relatively aerobic gut [34,35]. Despite this, as seen in other vespertilionid bats, Indiana bats host diverse and abundant *Clostridia* (Fig. 1a) [15]. *Clostridia* are a class of spore-forming, non-sulfate-reducing, Gram-positive bacteria that are predominately obligate anaerobes [15]. Facultatively anaerobic *Clostridia* clades (both commensal and pathogenic) may thrive in the bat gastrointestinal tract despite its limited anaerobic volume by using an intracellular strategy that is tolerant of limited concentrations of oxygen [14]. The most abundant *Clostridia* ASV shared 98.52% identity (by blastn) with two recently discovered segmented filamentous bacteria (SFB) isolates of mice and rats [36]. These related taxa are potent inducers of T helper 17 immune cells in their respective hosts [36]. SFBs are obligate anaerobic commensals that, in many mammals, spread from mother to offspring via spores [37,38], which could amplify their detection in the guano [39]. The role that these putative bat SFBs play in gut health will require additional research beyond the scope of this study [40,41]. There were also diverse *Clostridium sensu stricto* ASVs prevalent, which increased in relative abundance with the incidence of an *Eimeria* spp. parasite (Fig. 1b, c).

### 18S parasitome composition of Indiana bats, Missouri, Fall 2022

**Fig. 2.**
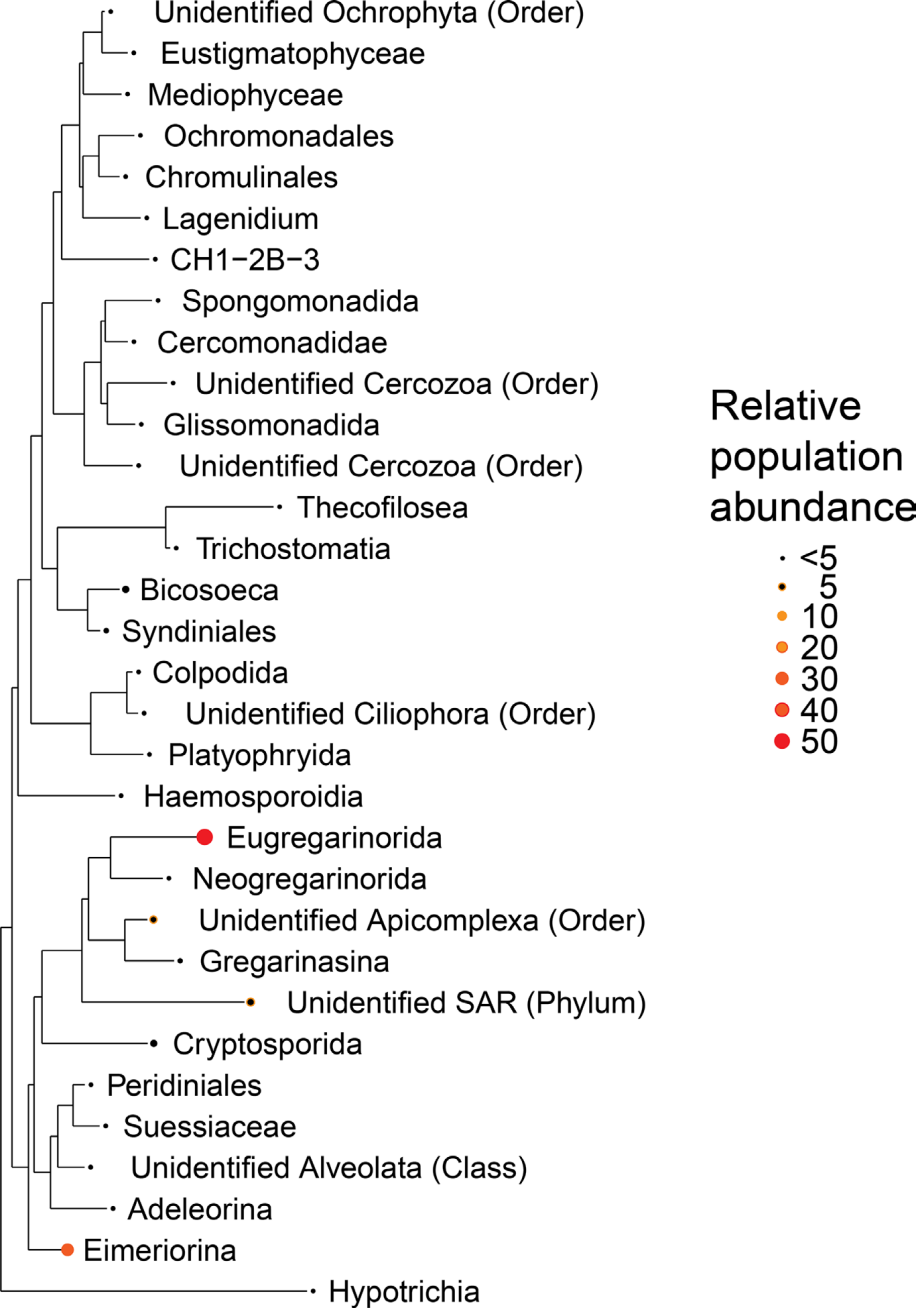
Maximum-likelihood phylogeny of protozoans in the guano of Indiana bats (*n*=56), Missouri, Fall 2022. 18S rRNA phylogeny of SAR supergroup ASVs, agglomerated by family. Points represent the relative population abundance of reads assigned to the taxonomic family in a pooled dataset of all Indiana bat faecal samples in the study.

Due to the mixed composition of guano, our 18S sequence data likely describe protozoans of bats, their arthropod prey and the plant or invertebrate diet of those prey [42]. Moreover, molecular detections could even be of micro-organisms that naturally infect ectoparasites [43] and are incidentally ingested [44] but pose little to no harm to our study species [42]. These complications – inherent to molecular surveillance of mixed sample types like faeces – should encourage careful interpretation of molecular diagnoses. Our initial 18S sequence read set included 10 987 958 reads assigned to 5671 ASVs. Filtering reads assigned to off-target clades removed 4697 ASVs, leaving 2 514 275 reads assigned to 974 SAR supergroup ASVs [36.4±2.5 (se) ASVs and 44 410±6040 (se) reads per sample]. Normalizing read counts reduced the total dataset to 791 SAR ASVs [mean 37.1±2.3 (se) ASVs per sample]. Tip agglomeration resulted in 256 SAR clades representing 31 known apicomplexan families (Fig. 2). Parasite diversity in guano was comparable at each site, and each SAR family had representative ASVs detected at both sites (data not shown). Most reads represented gregarine protozoans (e.g. Eugregarinorida), a large group of gut protozoans that only infect invertebrates and thus are likely derived from the arthropod diet of the bats [45,46]. We also detected ASVs representative of *Bicosoeca* [47] and *Adelorina* spp., which are protozoans related to the free-living protists but poorly described in the biomedical literature [48]. We detected a suspected vertebrate parasite of genus *Eimeria* [49], which shared >99% sequence identity with *Eimeria rioarribaensis* (GenBank accession No. AF307877.1) isolated from another species of vespertilionid bat [50,51]. *Eimeria* ASVs were detected in 24 of 56 guano samples [42.9% (95% CI: 29.7%, 56.8%)]. We did not detect a statistically significant difference in the prevalence of *Eimeria* across our two sites, with 10 of 31 [33.2% (95% CI: 15.8%, 48.7%)] samples positive from Lime Kiln and 14 of 26 [53.9% (95% CI: 33.4%, 73.4%)] samples positive from Pilot Knob.

*Eimeria* spp. parasites have been reported in >30 bat species [52] and are likely broadly prevalent given the ubiquity of *Eimeria* across diverse terrestrial vertebrate hosts [49,52]. *Eimeria* is a diverse and ubiquitous genus of coccidian protozoan parasites that cause mild to severe gastrointestinal disease in mammals, birds, amphibians and reptiles [53]. *Eimeria* has a direct life cycle with the intraspecific transmission of shed oocysts via the faecal-oral route [49,54]. Although the dynamics of how *Eimeria* persists in bat populations has not been systematically studied, *Eimeria* spp. are known to amplify via acute infections of immunologically naïve juveniles [55,56]. Low-intensity, subclinical *Eimeria* infections [56] may clear in time without issue or may develop into acute disease after a slow and incremental increase in intensity [49] if the host becomes stressed [57] or as a result of interactions with the microbiome and sympathetic co-infections [58,60].

*Eimeria* parasites cause coccidiosis in diverse hosts [49,53, 55, 56] and are a significant burden to cattle and domestic chickens [49,53]. *Eimeria* disease can present as acute intestinal coccidiosis with intestinal necrosis and inflammation, which results in severe diarrhoea that can be life-threatening [49], but infections can also be asymptomatic or self-limiting [52]. *Eimeria* infection results in inflammation, an increase in permeability of the gut epithelium and a reduction in efficiency of nutrient uptake [59].

Interpreting the relevance of the simple presence or absence of *Eimeria* to health should be done with care. Our best knowledge of *Eimeria*-associated illness is due to experimental infections of domesticated species [61,62], so we do not know the typical impact of the parasites on an arbitrary host. When studying endangered species, it is difficult to rely on extensive lethal sampling for more definitive histopathology [6]. However, an understanding of host-population-level associations between parasites and the microbiome can contextualize a diagnosis with indirect molecular evidence of the underlying pathophysiology [58,60, 62,64].

### Differentially abundant microbes in *Eimeria-*positive Indiana bats

**Fig. 3.**
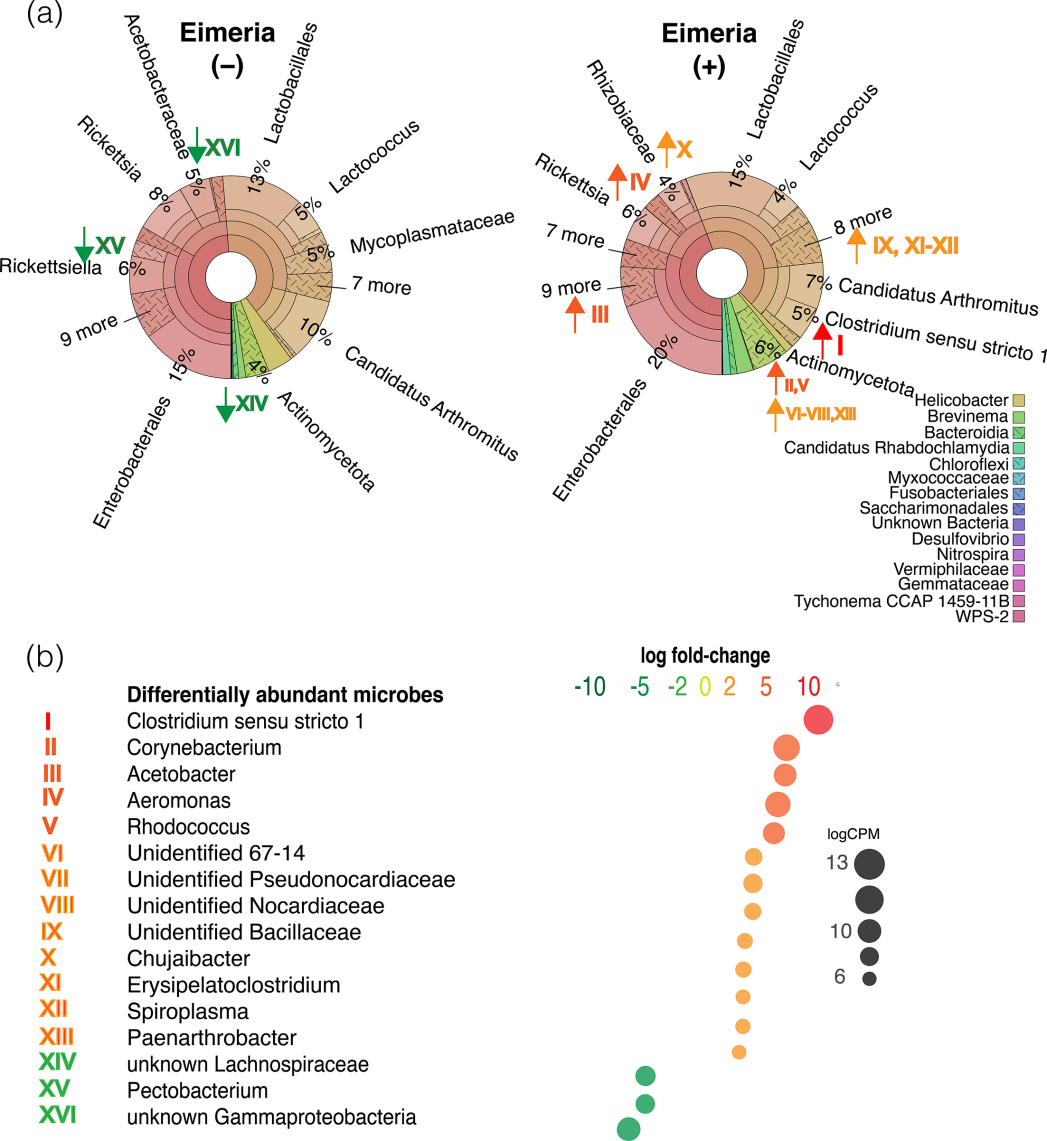
*Eimeria* status correlates with differential abundance of microbes in Indiana bats (*n*=56). (a) Krona plots representing the composition and relative read abundance of the 16S faecal microbiome of *Eimeria*-positive (*n*=32) and *Eimeria*-negative (*n*=24) Indiana bats. Roman numerals represent DAMs, and arrows indicate the direction of fold-change in *Eimeria*-positive bats. The inset key indicates names and Krona wedge colour of taxa present at ≤3% relative abundance. (**b) **Log_2_ fold-change (logFC) of differentially abundant microbes (DAMs) in *Eimeria-*positive relative to *Eimeria*-negative bats. Size represents log c.p.m. (logCPM) of the DAM. *P*-values were adjusted by Benjamini-Hochberg (*a*=1×10^−6^) to control the false discovery rate. The taxonomic position of the top 16 DAMs is plotted on Krona plots by a Roman numeral.

We tested for DAMs in *Eimeria*-positive bats, and the highest fold-change increase was seen for an unidentified *Clostridium sensu stricto* species, which contributes to shifts in overall composition with *Eimeria* (Fig. 3a). By blastn, the 16S ASV of this top DAM (Fig. 3b) has >97% identity with the *Clostridium perfringens* type strain (NR_121697.2) and 100% identity with a proposed new species isolated from human stool: *Clostridium massiliamazoniense* (NR_178427.1) [65]. *C. perfringens* is a species of pathogenic *Clostridium* responsible for necrotizing enteritis in broiler chickens with acute coccidiosis [58,60].

In acute coccidiosis, *Eimeria* species parasites invade the gut epithelium, damaging tissues and inducing dysbiosis in chickens (*Gallus gallus domesticus*) and cattle (*Bos taurus*) [55,58, 59, 61, 63, 64]. In experimental settings, *Eimeria* infection is characterized by a reduction of several commensal clades and a synergistic proliferation of necrotizing *Clostridium* species [58,60]. *Clostridium* species can invade and further damage epithelia, particularly in the heightened aerobic conditions that can arise due to *Eimeria*-associated lesions [15,60]. *Clostridium* species can respond to aerobic conditions by invading the host epithelium, where the typically anaerobic clade has mechanisms to survive despite low levels of oxygen and associated compounds [15]. Aerobic conditions can arise due to *Eimeria*-associated damage [15,60], and *Eimeria-*associated mucogenesis offers a growth advantage to *C. perfringens* in chickens [66]. The damaged gut epithelia can then foster additional opportunistic infections, which may associate with lesions or otherwise benefit from changes to the gut microenvironment with infections (and coinfections) of *Eimeria* and pathogenic *Clostridium* species [61].

In chickens, *Eimeria-*associated necrosis fosters an environment in which opportunistic pathogens flourish [49,53, 59]. For instance, the *Corynebacteriaceae* are commensal bacteria of mucous membranes that proliferate opportunistically in damaged tissue [67]. In chickens, increasing *Eimeria* dose results in an over-expression of *Corynebacterium* and other Actinomycetota through related processes [68]. The DAMs of this clade in *Eimeria-*positive bats could represent a similar phenomenon [69]. An ASV of the family *Lachnospiraceae* was the most downregulated DAM. This clade is believed to be predominantly commensal to vertebrates, although due to difficulty culturing the taxa [41], our broader, comparative understanding of its biology has lagged [70]. However, a reduction of commensal bacteria, including those of the *Lachnospiraceae*, is common in experimental *Eimeria* infections of chickens [58,59].

### Potential consequences of pathologic *Eimeria–Clostridium* coinfection to pre-hibernation bats

We sampled bats during the fall swarm at two large hibernacula, weeks before hibernation was expected to begin. Across our two sites, 43% of Indiana bats were infected with *Eimeria*. We identified associations between *Eimeria* and differential microbe abundance that parallels a suite of microbiome changes typical to pathologic infection with the parasite in other species. * Eimeria* infection was strongly associated with a large and significant increase in the relative abundance of *Clostridium* and diverse opportunistic pathogens and the reduction of a commensal clade [58,60,63, 64]. These changes could indicate *Eimeria-*associated necrosis in bats, although the evidence is not direct.

*Eimeria*-associated necrosis disrupts nutrient absorption in birds and in diverse mammals [49], which could be especially relevant to hibernating North American bats. Bats face extreme food deprivation in winter months and rely on fat reserves and minimal arousal to survive [71]. Since the invasion of *Pseudogymnoascus destructans* and the emergence of white-nose syndrome in North America, the challenges of hibernation have been put in sharp relief. North American bats prepare for a time of resource scarcity and extreme weather [71], during which their skin is vulnerable to topical and invasive infection by *P. destructans* [72]. White-nose syndrome kills bats by increasing the frequency of arousals during overwinter hibernation and sapping energy reserves [73]. The study of coinfections of *P. destructans* and other pathogens has been limited [74], but if Indiana bats are experiencing acute coccidiosis with a concomitant increase in necrotizing *Clostridium* [49,53, 55, 60] immediately prior to hibernation, they may be at a severe disadvantage [75].

More invasive research would be needed to confirm that the over-expression of *Clostridium* that we see with *Eimeria* infection in Indiana bats is correlated with tissue damage in the gut, as seen in other species [49,53, 55, 60]. Definitive associations between molecular detection and disease typically require pathogen isolation for *in vitro* or *in vivo* challenges of live cells or laboratory animals, paired with direct assessment of associated pathology [6]. Non-invasive monitoring of wildlife population health using multiplex metabarcode sequencing of faeces is an indirect method, but with insights from comparative microbiology, we can offer context to molecular surveillance data [76] even when establishing a population snapshot baseline in a rare, cryptic species like the Indiana bat.

## Supplementary material

10.1099/mgen.0.001358 Uncited Supplementary Material 1.

## Abbreviations

ASVs: amplicon sequence variants
DAMs: differentially abundant microbes
RDP: Ribosomal Database Project
SFB: segmented filamentous bacteria
SRA: sequence read archive
SRR: sequence read runs
